# Returning to Work and Cost-Effectiveness After Lumbar Facet Cryodenervation Among Patients with Chronic Low Back Pain

**DOI:** 10.3390/jcm15051825

**Published:** 2026-02-27

**Authors:** Michał Krakowiak, Julia Stelmach, Jarosław Dzierżanowski, Tomasz Borusiński, Piotr Zieliński

**Affiliations:** 1 Department of Neurosurgery, Medical University of Gdańsk, 80-210 Gdańsk, Poland; 2 Polish Scientific Circle of Neurosurgery, Medical University of Gdańsk, 80-210 Gdańsk, Poland; 3 Neurosurgery Department, Wojewódzki Szpital Specjalistyczny, 76-200 Słupsk, Poland

**Keywords:** occupational disability, work incapacity, work reintegration, workplace productivity

## Abstract

**Background/Objectives**: Low back pain (LBP) is a leading cause of disability and work absenteeism worldwide. Lumbar facet joint degeneration is a common source of chronic LBP, and when conservative treatment fails, interventional procedures may be indicated. Cryodenervation is a minimally invasive option that remains less extensively studied. This study aims to evaluate clinical outcomes, cost–utility, and return-to-work rates following lumbar facet joint cryodenervation. **Methods**: A retrospective study included 42 professionally active patients treated with lumbar facet joint cryoablation between 2020 and 2022 at a tertiary neurosurgical center. All patients had facet-mediated LBP confirmed by a positive diagnostic medial branch block. Pain (VAS), disability (ODI), and work status were assessed before and after treatment. ODI scores were converted to SF-6D utilities to estimate quality-adjusted life years (QALYs). Cost data were obtained from institutional records. **Results**: Mean ODI improved from 48.5 ± 12.8 to 36.6 ± 17.8, and mean VAS from 7.0 ± 1.7 to 3.8 ± 2.0. Mean SF-6D increased from 0.53 to 0.59, corresponding to a gain of 0.0103 QALYs over four months (annualized 0.0309). The mean procedure cost was 1905 PLN, resulting in approximately 185,000 PLN per QALY, which is within the national cost-effectiveness threshold. Overall, 58.5% of patients returned to work, with the highest rate in those aged 30–39 years (83.3%). **Conclusions**: Lumbar facet cryoablation provides meaningful pain relief and functional improvement at a favorable cost-effectiveness profile. Younger patients show higher return-to-work rates. Larger prospective studies are required to confirm these findings.

## 1. Introduction

Low back pain (LBP) is a leading worldwide issue affecting an average 31% of the population [1]. At present, there is no established gold standard for the diagnosis of LBP [2]. Neither physical examination findings nor elements of the patient’s medical history are pathognomonic or allow for a definitive determination of pain origin [3]. When conservative management fails to provide adequate symptom relief, interventional treatment options may be considered, including procedures targeting the nerve branches innervating the affected facet joints [4]. Among these, thermal ablation is the most widely used and extensively studied technique and has been reported in the literature to provide meaningful pain relief in selected patients. Cryoneurolysis, although supported by more limited scientific evidence [5], has also been described as a potentially effective analgesic intervention and is increasingly considered as an alternative treatment option for LBP [6,7,8].

In European countries and Israel, LBP occurred in 19% of adults in moderate to severe intensity influencing the quality of social and work life [9]. The peak incidence of LBP is observed between the ages of 35 and 55 [10]. According to data from 1993, in the UK, LBP was the most common cause of absence from work, achieving a total of 52 million lost working days [11]. In Sweden, the mean total cost per LBP episode, defined as direct healthcare costs and indirect costs in terms of sick leave and early retirement, was estimated at €2753. The overall economic burden caused by LBP in Sweden of all LBP episodes that started in 2011 estimates around €740 million, or €78 per capita [12]. LBP has the highest rate of years lived with disability (YLD), contributing to over 146 million YLDs in 2013 with a 61% increase compared to 1990 [13]. In emergency departments, LBP represents 5.9% of patients with the admission of 16% and this number is still increasing over time [14]. Lumbar facet joints are a common source of LBP. Clinical presentation is defined as back pain radiating to uni- or bilaterally to buttocks; additionally, it can affect the groin and thighs, stopping above the knee. When conservative treatment proves ineffective, invasive methods remain an option, including lesioning of the articular nerve branches supplying the affected joint [15]. Thermal ablation, being the most widespread and commonly used technique, is described in international literature as a relatively safe and effective procedure. Cryoablation, on the other hand, has considerably weaker scientific support [5], yet its analgesic efficacy has also been documented, and it is recognized as an established method for the treatment of LBP [5,7,8,16]. There is an increase in lumbar procedures, and the rate of ablation procedure performed grew 9.7% annually [17]. We aim in this study to evaluate the clinical outcomes, cost–utility, and return-to-work rates of lumbar facet joint cryodenervation in patients with chronic facet-mediated low back pain, and compare these findings within the broader socioeconomic burden of low back pain worldwide.

## 2. Materials and Methods

A retrospective study was conducted on patients who performed cryodenervation of lumbar facet joints between 2020 and 2022 in the Neurosurgical Deparment at the Department of Neurosurgery, University Clinical Center in Gdańsk. The study received approval from the Clinical Research Department of the University Clinical Center in Gdańsk number 233/2024. In Poland, the reporting of medical procedures for claims and reimbursement with the National Health Fund (NFZ) is conducted using the national ICD-9-PL CM procedure classification. Therefore, procedures were identified using the ICD-9-PL CM (03.96- Percutaneous denervation of facet) classification system, and economic data were obtained from the SGA company via the MyHospital platform(Warsaw, Poland) [18,19].

The inclusion criteria consisted of patients with LBP consistent with the accepted definition [20], persisting for more than 7–12 weeks and meeting the criteria for chronic pain that did not respond to conservative treatment [21]. LBP had to be the primary complaint, with no clinical signs of nerve root compression or spinal canal stenosis. Pain was required to originate from the L4–L5 level, confirmed by the examining physician based on the patient’s medical history, physical examination, imaging findings, and diagnostic block results.

Eligibility for cryodenervation was based on the response to a diagnostic medial branch block (MBB). All patients underwent a single diagnostic MBB performed in a neurosurgical outpatient setting. A total volume of 2–3 mL of 1% lidocaine was administered to each medial branch of the dorsal ramus innervating the L4–L5 facet joint. No long-acting local anesthetics were used. The procedure consisted of a single diagnostic block; no dual comparative blocks were performed. No placebo-controlled or sham procedures were used.

Pain intensity was assessed using the Numeric Rating Scale (NRS, 0–10). Baseline NRS was recorded immediately prior to the block, and reassessment was performed 10–20 min after anesthetic administration, corresponding to the expected onset of lidocaine action.

A positive diagnostic response was defined as a reduction in pain intensity of ≥50% on the NRS compared to baseline, consistent with the criteria proposed by Bogduk [22].

Only professionally active patients with documented work incapacity due to LBP were included. A postoperative follow-up visit performed 4–6 months after the procedure was mandatory.

The exclusion criteria were as follows: prior lumbar spinal neurosurgical intervention; presence of “red flag” symptoms or neurological findings suggestive of pathology requiring alternative neurosurgical management [23]; imaging evidence of degenerative or structural abnormalities potentially responsible for lumbar pain (e.g., acute disk herniation, spondylolisthesis, scoliosis, vertebral fracture, infection, or suspected neoplastic disease); incomplete medical documentation at the time of hospital admission or postoperative follow-up preventing inclusion in statistical analysis; and patients lost to follow-up or not evaluated in the neurosurgical outpatient clinic after the procedure.

Cryoanalgesia was performed using the CRYO-S^®^ Painless device (Metrum Cryoflex, Łomianki, Poland), routinely utilized at the Department of Neurosurgery, University Clinical Center. The system operates with carbon dioxide (CO_2_) as the cryogenic agent, achieving temperatures of up to −70 °C. Round-tip cryoprobes with a diameter of 1.5 mm (model A-13/120/R/RF) were used in all procedures. All interventions were performed in the operating theater setting, under sterile conditions, under fluoroscopic guidance and with local anesthesia with the use of 1% lidocaine.

The operative technique involved positioning the C-arm to obtain the characteristic “Scotty dog” oblique projection of the lumbar vertebrae. This projection was used to accurately identify the target point for probe placement at the level of the medial branch of the dorsal ramus. Final probe positioning was verified using neurostimulation to confirm appropriate sensory response and exclude motor involvement.

Each cryoablation cycle consisted of two freezing periods of 120 s each, reaching a target temperature of −70 °C at the probe tip. For denervation of the L4–L5 facet joint, cryodenervation was performed on the medial branch arising from the L3 dorsal ramus and the medial branch of L4, in accordance with the standard anatomical innervation pattern.

The following data were collected: patient age, smoking status, type of occupation (sedentary or physical), and duration of sick leave prior to the procedure, polish questionnaire Oswestry Disability Index (ODI) version 2.1 [24], back pain Visual Analog Scale (VAS). Follow-up data were collected at a control outpatient department visit 4–6 months after the procedure.

Functional disability assessed using ODI was converted into health utility values using a validated mapping approach. SF-6D utility scores were estimated according to the following equation proposed by Carreon. The applied ODI–SF-6D regression model has been previously reported to yield a correlation coefficient of 0.82, explain 67% of the variability in SF-6D utility values, and demonstrate a root mean square error (RMSE) of 0.078 [25]. SF-6D utility = 0.78275 − (0.00518 × ODI score). Quality-Adjusted Life Years (QALYs) were used as the primary measure of health benefit to quantify the combined effect of changes in health-related quality of life and survival. Health utility values were derived from the SF-6D index, calculated pre- and post-intervention. The QALY gain associated with the procedure was estimated assuming a linear change in utility over the observation period, using the trapezoidal rule, as recommended in economic evaluation guidelines [26]. The incremental QALY gain (ΔQALY), representing the effect of the intervention itself, was defined as follows:

$$\Delta QALY=\frac{\left(U_{post}-U_{pre}\right)}{2}\times T\;$$

- $U_{pre}$ and $U_{post}$ represent the utility values before and after the procedure, respectively.
- T is the time between assessments, expressed in years.

Given the 4-month follow-up, T=4/12=0.333 years was used for all calculations. Annualized QALY gains were derived by extrapolating this value to a 12-month period under the assumption of stable post-treatment utility.

### 2.1. Economic Analysis Framework

The economic evaluation was conducted from the perspective of the public healthcare payer (National Health Fund, NFZ). Only direct medical costs were included in the analysis. These costs were derived from the hospital reimbursement tariff (JGP system) and reflected the total real hospitalization cost per patient, including procedural, operating theater, personnel, and inpatient care components.

Indirect costs, including productivity loss, sick leave compensation, employer costs, and patient out-of-pocket expenses, were not included in the economic analysis. Return to work (RTW) was analyzed exclusively as a clinical outcome and was not incorporated into cost calculations. Sick leave costs were not calculated and were not included in the economic model.

### 2.2. Statistical Analysis

Statistical analyses were performed using PQStat version 1.8.0 (PQStat Software, Poznań, Poland). The distribution of continuous variables was assessed using the Shapiro–Wilk test. As most variables did not meet the criteria for normal distribution, non-parametric statistical methods were applied. Analyses related to return-to-work outcomes were performed by comparing patients who returned to work with those who did not return to work after the procedure. For these between-group comparisons of continuous variables, the Mann–Whitney U test was used. Categorical variables were analyzed using Fisher’s exact test, as appropriate. Descriptive statistics are presented as arithmetic mean, median, and standard deviation. The level of statistical significance was set at *p* < 0.05 (two-sided) for all analyses. No adjustment for confounding was performed beyond univariate comparisons.

## 3. Results

**Figure 1:**
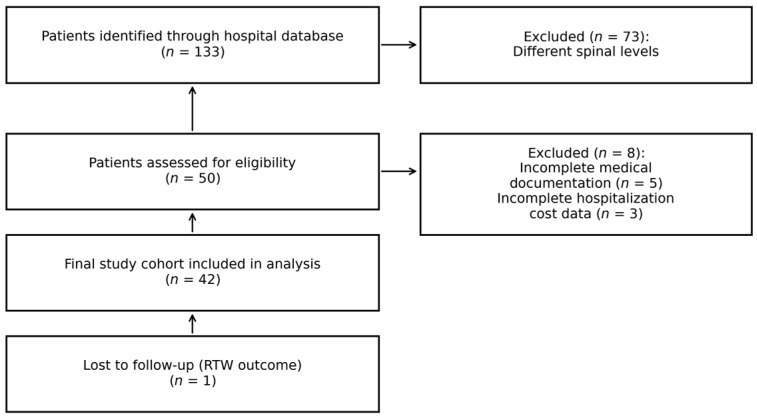
Flow diagram illustrating patient selection process.

A total of 133 patients was screened, resulting in a final study cohort of 42 patients after application of the inclusion and exclusion criteria. One patient (*n* = 1) was lost to follow-up with respect to return-to-work outcomes (Figure 1).

The mean patient age was 49.8 years (SD = 11.8), and 58.5% were female. Overall, 53.7% of patients were active smokers, and the mean BMI was 26.1 kg/m^2^ (SD = 7.8). The mean preoperative ODI score was 48.5 (SD = 12.8), which improved to 36.6 (SD = 17.8) postoperatively. The mean VAS score for pain decreased from 7.0 (SD = 1.7) before the procedure to 3.8 (SD = 2.0) after the procedure. The mean duration of sick leave prior to the procedure was 41 days (median = 30, SD = 30.7), with no significant differences observed between gender or smoking status.

**Figure 2:**
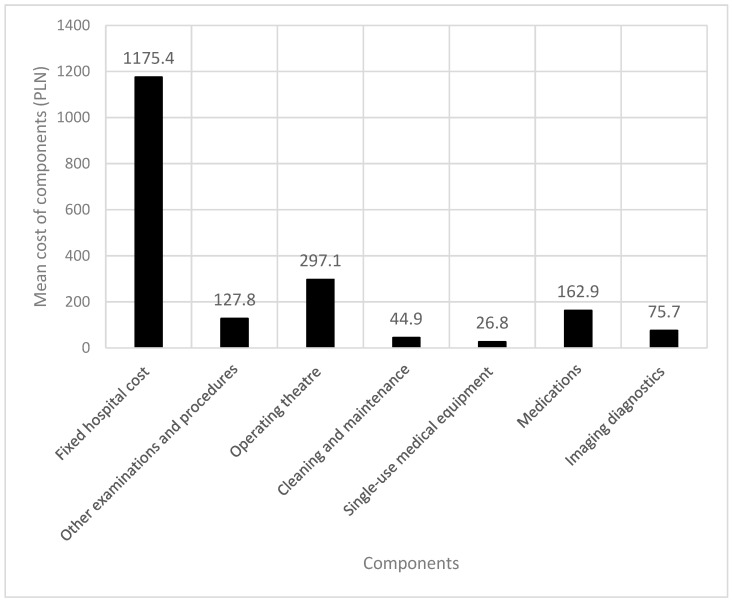
Mean cost structure of lumbar facet joint cryoablation per patient.

The mean preoperative SF-6D utility score, derived according to the applied conversion algorithm, was 0.5312 (SD = 0.07), and the mean postoperative score was 0.5929 (SD = 0.09), corresponding to a mean improvement of +0.062. The mean QALY gain during the 4-month observation period (0.333 years) was estimated at 0.0103 QALY per patient, equivalent to approximately 3.8 additional days of life in full health. When annualized, this corresponds to an estimated gain of 0.0309 QALY per patient per year. The total cumulative QALY for the entire study cohort was 7.68. The mean total cost per procedure was 1905.2 PLN (SD = 1465.8). The detailed breakdown of cost components is illustrated in Figure 2.

The total cumulative cost for all 42 procedures was 78,114.85 PLN, corresponding to a mean cost of 1905.24 PLN per patient. Based on the mean QALY gain of 0.0103 per patient during the 4-month observation period, the estimated cost of gaining one QALY was 184,975 PLN, or 61,658 PLN when annualized.

**Figure 3:**
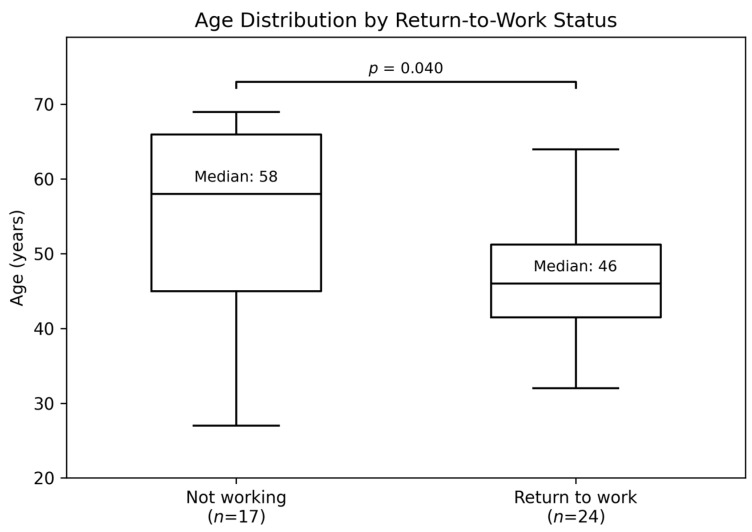
Age distribution by return-to-work status after lumbar facet joint denervation.

During the follow-up visit after cryoneuroablation, 17 patients (41.5%) did not return to work, whereas 24 patients (58.5%) resumed their occupational activity. In univariate analyses, gender, type of work, smoking status, and duration of pre-procedural sick leave were not significantly associated with return-to-work status. Age was a statistically significant factor (Figure 3).

The return-to-work status divided into age groups is presented in Table 1.

## 4. Discussion

Costs generated by LBP have been evaluated in various studies, showing their great burden on healthcare systems. Therefore, it is important to provide treatments that are cost-effective. In Switzerland, direct medical costs reached 6.1% of the total healthcare expenditure, estimated at €2.6 billion. Data from Sweden showed the total cost of LBP to be almost 2 billion € in 2001 [27,28,29]. In a study from Australia, the total 5-year period non-serious LBP episode costs of inpatient and emergency department were $36.7 million [30]. In high-income countries, the costs of LBP per patient were $9231, and in low-income countries, the total costs were $2.2 billion per population; in US, the costs were $1226.25 per patient annually [31,32]. In Poland, as of 2024, Social Insurance Institution (ZUS) recorded 27.4 million sick leave medical certificates for temporary incapacity for work, accounting for a total of 290.0 million days of sickness absence, which was 14.65 days per one insured individual [33]. In 2024, dorsalgia accounted for 5.0% of total sickness absence days for men and 2.9% for women.

The cost-effectiveness of various LBP treatments such as interdisciplinary rehabilitation, acupuncture, spinal manipulation, and cognitive–behavioral therapy for sub-acute or chronic LBP has been evaluated in a meta-analysis published by Lin et al., providing evidence for their cost-effectiveness [34]. Adding acupuncture to physician care showed the incremental cost-effectiveness ratio to be €10,526 per QALY [35]. In years 2007–2016, in USA, the cost per 100,000 lumbar facet injections rose from $257,280 to $396,580 within the time period. The cost per 100,000 radiofrequency ablations increased from $94,570 in 2007 to $266,680 in 2016, accounting for 12.2% annual increase [17].

In contrast, a study conducted in Thailand reported that radiofrequency ablation was not cost-effective compared with conservative treatment. The estimated ICERs were I$99,267/QALY and I$52,380/QALY over 16- and 28-month observation periods, respectively. The authors concluded that, within the Asian healthcare system context, radiofrequency ablation for LBP was not a cost-effective intervention [36]. In the MINT study, radiofrequency denervation combined with an exercise program was compared with a standardized exercise program. The maximum probability of cost-effectiveness was 0.65 at a willingness-to-pay threshold of €30,000 per QALY, indicating that the intervention was not cost-effective from a societal perspective for patients with LBP in the Dutch healthcare [37]. In knee osteoarthritis, cooled radiofrequency ablation was compared with intra-articular hyaluronan injections and was associated with a favorable cost-effectiveness profile, yielding a QALY gain of 0.020 at an incremental cost of US $1707, corresponding to an ICER of US $84,392 per QALY over a 6-month observation period [38].

In accordance with the polish law and the Agency for Health Technology Assessment and Tariff System, the threshold for the cost of obtaining an additional QALY or, where it is not possible to determine such cost, the cost of obtaining an additional life year is established at a level equal to three times the Gross Domestic Product per capita which was 217 641 PLN in years 2020–2022 [39].

Within the applied analytical framework, the estimated cost per QALY gained through cryoablation fell below the willingness-to-pay threshold defined by Polish health economic regulations. However, these findings should be interpreted with significant caution, as the economic evaluation was based on short-term follow-up and indirect utility estimation [40]. Such preliminary indicators provide a valuable baseline but do not represent a definitive long-term cost-effectiveness conclusion. This magnitude of benefit is comparable to other minimally invasive interventions aimed at pain reduction [5,7,41]. Kočan achieved a reduction in ODI from 59.4 to 46.1 with the use of cryoablation, showing that radiofrequency ablation, cryoablation, and endoscopic denervation treatment provide significant and sustained improvements in LBP and functional status over the two-year follow-up [41]. Randomized prospective trials comparing cooled versus traditional radiofrequency ablation of medial branches for lumbar facet joint pain noted no significant difference between the methods of achieving comparable results after one diagnostic block [41,42]. Given the relatively low procedural cost, such an effect size may still translate into a favorable cost-per-QALY ratio, though further validation is required. Data indicate that approximately 68.2% of patients return to work within one month after an episode of LBP, increasing to 93.3% after six months or more [43]. In Poland, in 2022, the average daily amount of sickness benefit financed by the Social Insurance Fund (FUS) amounted to PLN 103.44, reflecting a 7.9% increase compared with 2021 [44].

In our study, the highest proportion of patients returning to work after facet joint denervation was observed in the 30–39 year age group (83.3%), with only 30% of patients aged 60–69 years resuming work. Age was the only variable significantly associated with return to work in univariate analysis. In the present cohort, gender, type of work, smoking status, and the duration of pre-procedural sick leave were not significantly associated with return-to-work outcomes. These findings suggest that, within the limits of univariate analysis, no statistically significant associations were observed.

Several factors have been identified as predictors of failure to return to work, including higher resting pain intensity or subjective perception of constant back strain during occupational activities. However, during the 24-month follow-up period, being younger than 41 years was identified as the only significant predictor of not returning to work [45]. Evidence concerning the relationship between the timing of intervention and occupational outcomes remains inconsistent. Okurowski et al. reported a weak but positive association between earlier referral and shorter duration of disability, whereas a study by Shaw has demonstrated that early referral is linked to better employment outcomes and a higher probability of return to work [46,47]. In contrast to previous studies demonstrating that occupational type (especially blue-collar work) and job satisfaction are significant predictors of return to work, our analysis did not reveal any association between the type of work and the likelihood of returning to employment [48]. Total expenditures related to sickness absence in 2024 amounted to 31,039.7 million PLN, representing a 16.1% increase compared to the previous year [33]. Van der Wurf analyzed the costs of sick leave among employees in the Netherlands related to LBP between 2015 and 2017, using data from occupational physicians’ records of medical absences. In total, 7901 LBP episodes among 7161 unique employees were examined. The data indicated that the mean duration of a sick leave episode was 129.42 days, with an average cost of €16,191 per episode. The total extrapolated cost of sick leave due to LBP in 2017 was estimated at €244.7 million [49]. From an occupational health perspective, cryoablation may contribute to reduced work absenteeism and improved occupational reintegration among patients with chronic facetogenic LBP and potentially reduce the burden on the Polish healthcare system.

## 5. Future Directions

Future studies should include larger, prospective cohorts with extended follow-up to confirm the long-term durability and cost-effectiveness of lumbar facet joint cryoablation. The inclusion of a control group is essential to establish causal inference. Incorporating standardized occupational and psychosocial outcome measures will help identify key predictors of successful return to work and sustained functional recovery.

## 6. Limitations

Several methodological limitations should be acknowledged. First, the retrospective, single-center design and the absence of a control or comparison group inherently limit the ability to draw causal conclusions regarding clinical and economic outcomes. Secondly, QALY estimates were derived indirectly through ODI-to-SF-6D mapping and were based on a short-term (4-month) follow-up. This approach assumes health utility stability over time and may fail to capture potential attenuation of the treatment effect or the influence of unmeasured confounders, such as concurrent rehabilitation or psychosocial factors. Therefore, these economic findings should be considered exploratory and preliminary rather than definitive. No formal sensitivity analysis was conducted.

## 7. Conclusions

Cryoablation demonstrated short-term improvements in pain and quality of life within a payer-based economic framework, with preliminary indications of favorable cost–utility. The study demonstrates improvement in pain and function and demonstrated preliminary cost–utility within a short-term payer-based framework. Future prospective and long-term studies are warranted to confirm sustained functional recovery and to better define occupational and psychosocial predictors of successful return to work.

## Figures and Tables

**Table 1:** Return-to-work status across age categories in patients after facet joint denervation.

Age Group (Years)	Did Not Return (%)	Returned to Work (%)	Number of Patients (*n*)
20–29	50	50	2
30–39	16.7	83.3	6
40–49	33.3	66.7	18
50–59	40	60	5
60–69	70	30	10

## Data Availability

The raw data supporting the conclusions of this article will be made available by the authors on request.

## Abbreviations

The following abbreviations are used in this manuscript:

LBP	Low back pain
ODI	Oswestry Disability Index
SF-6D	Short Form 6 Dimensions
VAS	Visual analog scale
QALY	Quality-Adjusted Life Years
YLD	Years lived with disability
NFZ	National Health Fund
RTW	Return to work
